# Cardiovascular disease prevalence in type 2 diabetes – an analysis of a large German statutory health insurance database

**DOI:** 10.1186/s12889-021-10381-z

**Published:** 2021-02-09

**Authors:** Maximilian Gabler, Silke Geier, Lukas Mayerhoff, Wolfgang Rathmann

**Affiliations:** 1 Boehringer Ingelheim, Ingelheim am Rhein, Germany; 2 Former employee of Elsevier Health Analytics, Berlin, Germany; 3 Praxis für Integrative Medizin, Hamburg, Germany; 4 Institute for Biometrics and Epidemiology, German Diabetes Center, Leibniz Center for Diabetes Research at Heinrich Heine University, Düsseldorf, Germany

**Keywords:** Secondary data analysis, Epidemiological study, Prevalence figures, Type 2 diabetes mellitus, Cardiovascular disease

## Abstract

**Background:**

The aim of this study was to determine the prevalence of cardiovascular disease in persons with type 2 diabetes mellitus (T2D) in Germany.

**Methods:**

A claims database with an age- and sex-stratified sample of nearly 4 million individuals insured within the German statutory health system was used. All patients aged ≥18 years with T2D documented between 1 January 2015 and 31 December 2015 and complete retrospective documentation of ≥5 years (continuous enrollment in the German statutory health system) before 2015 were selected based on a validated algorithm. Cardiovascular disease (CVD) events were identified based on ICD-10 and OPS codes according to a previous clinical study (EMPA-REG OUTCOME trial).

**Results:**

The prevalence of T2D in Germany in 2015 was 9.9% (*n* = 324,708). Using a narrow definition of CVD, the 6-year observation period prevalence of CVD was estimated as 46.7% [95% CI: 46.52%;46.86%]. Applying a wider CVD definition, the proportion of T2D patients who showed a history of CVD was 57.1% [95% CI: 56.9%;57.24%]. The prevalence of CVD in patients with T2D ranged from 36.3 to 57.1%, depending on the observation period and definition of CVD.

**Conclusions:**

The results underline the need for a population-based registration of cardiovascular complications in T2D.

## Background

Elevated blood-glucose concentrations in undiagnosed or poorly controlled type 2 diabetes (T2D) cause damage to blood vessels and peripheral nerves, resulting in a higher risk of cardiovascular diseases (CVD) like myocardial infarction, stroke, chronic kidney disease, blindness, and amputations [1–3]. These complications lead to a decreased life expectancy and reduced quality of life and are associated with high healthcare costs [2, 4, 5]. At the age of 60 years, a history of diabetes and stroke or myocardial infarction is associated with 12 years of reduced life expectancy and a history of all three of these conditions is associated with 15 years of reduced life expectancy [6]. Recent population-based prevalence estimates of T2D in Germany are in the range of 10% [7, 8]. Epidemiologic data on specific subpopulations of patients with T2D, however, are limited, with particularly few data available on the prevalence of manifest CVD.

The period of investigation – among other factors – may have a significant impact on estimates of CVD prevalence in T2D [9]. The aims of this explorative study were to provide epidemiologic data for persons with T2D in Germany with a history of CVD and to quantify the influence of different observation periods and algorithms on CVD prevalence estimates in T2D.

To estimate the possible impact of EMPA-REG OUTCOME, a recent trial (2010–2015) that demonstrated reductions in CV mortality in patients with T2D and established CVD treated with empagliflozin in addition to standard care [10–12], the definition of CVD used in the underlying primary analysis was aligned to the inclusion criteria of this trial [13]. The included criteria comprised thus cerebrovascular disease and stroke, angina pectoris and ischemic heart disease and arterial occlusive disease, and furthermore procedures of revascularization, coronary bypass or stent.

## Methods

### Data sources

This analysis was based on an anonymized dataset including longitudinal claims data of approximately 6.7 million persons, comprising approximately 10% of the German statutory health insurance (SHI) population between 2010 and 2015 [14]. For this study, a subset of nearly 4 million persons per year, representative of the German population in terms of age- and sex-distribution (as of 31 December 2013), was used [15].

The database includes information about the utilization of health care services on an individual level. The data included information on demographics (including mortality), ambulatory services and ambulatory diagnoses, hospitalization, including dates of admission and discharge, diagnoses and procedures, supply of reimbursed drugs by pharmacies (including the date of prescription), reimbursed remedies and aids as well as information on inability to work and disability.

The Institut für Angewandte Gesundheitsforschung (InGef) database used for this study has been described in detail earlier [16]. External validation of the database showed good consistency with the German population in terms of morbidity, mortality and drug usage. The proportion of men and women was similar in the database compared to the German population (49.0% men and 51.0% women), but the population included in the database is slightly younger (mean 40.4 vs 43.7 years). The proportion of SHI insured persons living in the eastern part of Germany was lower in the InGef database (10.1% vs 19.7%). There was good accordance with German reference data with respect to hospitalization rates, overall mortality rate and prescription rates for the 20 most often reimbursed drug classes, with the overall burden of morbidity being slightly lower in the InGef database. Only 2 % of individuals were lost to follow-up over the study period. Hence, the high validity, the comprehensive character as well as the low drop out rate of insured individuals were making the database a suitable and unique source for longitudinal epidemiological analyses. The InGef database has been accepted multiple times by health technology assessment (HTA) authorities in Germany as a valid data source [17, 18].

Due to data protection requirements, all analyses were conducted on the premises of InGef in Berlin, an SHI-associated institute, according to a predefined study protocol.

### Study population

The study population selected for this study included all adult patients (age ≥ 18 years) who had continuous enrollment in SHI between 1 January 2010 and 31 December 2015. Then, all persons with a diagnosis of T2D or glucose-lowering treatment between 1 January 2015 and 31 December 2015 were selected, including those who died after a T2D diagnosis in 2015. The algorithm to identify patients with T2D was based on the approach of the University of Cologne (PMV-Forschungsgruppe) as applied in several previous outcome research projects [4, 19–21]. International classification of diseases (ICD)-based diagnoses comprised assured ambulatory diagnoses as well as hospital main or secondary discharge diagnoses. Following this definition, T2D was defined as:

1. At least one prescription of a non-insulin glucose-lowering agent (Anatomical Therapeutic Chemical (ATC) classification code A10B) in at least two quarters in 2015 or
2. At least one prescription of a glucose-lowering agent (ATC code A10) plus at least one documented ICD-10 GM diagnosis of E11 in 2015 (ambulant ‘gesichert’, hospital main or secondary diagnosis) or
3. At least one prescription of a non-insulin glucose-lowering agent (ATC code A10B) plus at least one determination of blood glucose/ glycated hemoglobin (HbA1c) levels (Einheitlicher Bewertungsmaßstab (uniform evaluation standard, EBM) 32,025, 32,057, 32,881, 32,094) by the prescribing physician in the prescription quarter or
4. Repeatedly documented ICD-10 GM diagnoses of E11 in at least three out of four quarters in 2015 (ambulant ‘gesichert’ or hospital).

### Definition of cardiovascular disease

According to the criteria pre-specified in the EMPA-REG-OUTCOME trial, we defined patients who show a history of CVD to be a subpopulation of the T2D patients [10]. These criteria were translated into ICD-10 and OPS (Operationen- und Prozedurenschluessel, the national classification system of operations and clinical procedures in Germany) codes and the selected codes were then used to identify patients with a history of CVD within the study population. We aimed to include the most specific codes and leave out those not fully matching the EMPA-REG-OUTCOME inclusion requirements. The following ICD codes have been included as selection criteria identifying CVD: cerebrovascular disease and stroke (ICD-10 I63.−/I64.-), angina pectoris and ischemic heart disease (ICD-10 I20.0, I21-I22, I24-I25 excluding I25.3 / .4 / .10 / .19), revascularization (OPS 5–361, 5–362, 5–363), coronary bypass / stent (OPS 8–836, 8–837, 8-84x), and peripheral arterial occlusive disease (ICD-10 I73.9 / I70.2x). The inclusion criteria of the EMPA-REG OUTCOME trial did not contain several common CVDs such as chronic heart failure (CHF) thus (esenting a narrow approach of defining CVD. This narrow approach was used for the primary analysis of this study.

To assess the impact of variations of CVD definitions on the prevalence estimates, additional analyses were carried out. The broader definition of CVD included in addition to the above-mentioned codes, additional ICD codes of intracranial hemorrhage (I60 – I64), additional codes of ischemic heart disease (I20-I25) as well as chronic heart failure (I50). To estimate the effect of different observation intervals, we performed several sensitivity analyses, covering different time intervals. We also investigated how excluding OPS procedure codes from the inclusion criteria impacted the results; this sensitivity analysis was performed for the broad CVD definition excluding CHF using a whole six-year observation period.

### Statistical methods

Outcomes are reported descriptively, as absolute and relative frequencies with upper and lower bounds of 95% confidence intervals (CI) where appropriate. SAS 9.4. (SAS, Cary) was used to evaluate data and to compute descriptive statistics including arithmetic means or proportions and corresponding 95% CI for the variables of interest. This study was planned and executed following best practice guidance for secondary data analyses [22, 23]. The database quality management comprised defined data collection, management, and verification processes, including quality control processes and documentation of quality control steps.

## Results

The overall 2015 one-year prevalence in the InGef database for some common cardiovascular conditions are summarized in Table 1.

**Table 1:** One-year prevalence of common cardiovascular conditions and risk factors in the InGef database

Cardiovascular Condition (ICD-10-GM Code)	Number of Patients	Percentage
Essential Hypertension (I10)	1,182,757	30.5
Chronic Heart Failure (I50)	174,932	4.5
Stroke	63,447	1.6
Peripheral Vascular Disease	51,688	1.3
Acute Myocardial Infarction	32,598	0.8
Chronic Kidney Disease stage 5	7876	0.2

**Fig. 1:**
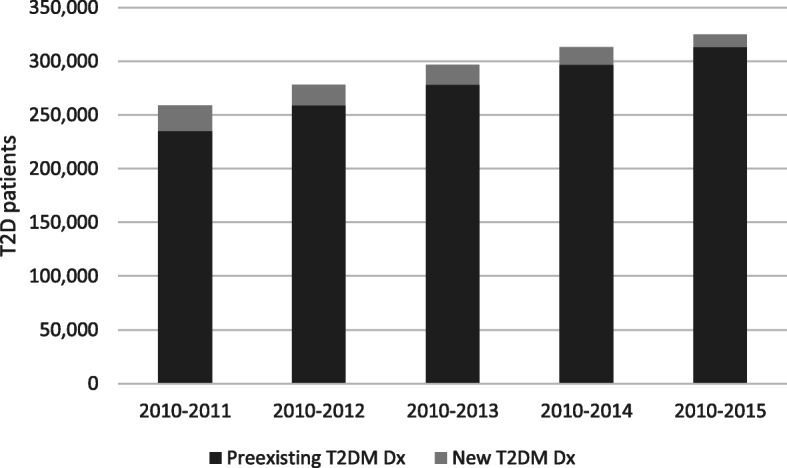
Incidence and prevalence of T2D. Note: Calculations are based on the “narrow” definition as explained in the Methods section. T2D = Type 2 diabetes, Dx = Diagnosis

A total of 3,229,909 were classified as adults in 2015 and had a continuous enrollment between 2010 and 2015. Out of these individuals, a total of 324,708 persons with T2D were identified as the prevalent base population in this study (cf. Figure 1). 8306 additional persons received a glucose-lowering drug treatment and had a diagnosis of “unspecific diabetes” (E14) but no “T2D diagnosis” (E11). These persons have not been included so that the study population comprises persons with definite T2D diagnosis only.

Table 2 shows the characteristics of individuals with a diagnosis of T2D in 2015. There were slightly more men (51.4%) than women. The mean age was 70.0 years with females being slightly older than males (71.8 years vs. 68.2 years). Almost 60% of the patients were between 60 and 80 years old, while only 1.5% of all patients with T2D were younger than 40 years.

**Table 2:** Flow of selection and patient characteristics of 324,708 individuals with a diagnosis of T2D in 2015

Parameter	Result
Number of patients in age and gender stratified database 2015	3, 876,632
and age ≥ 18 at 01.01.2015	3,296,948 / 100%
and observable between 2010 and 2015	3,229,909 / 98.0%
and diagnosis of T2D in 2015 - > Study population	324,708 / 9.9%
Female gender: Of study population / of all female insurants	48.6% / 9.3%
Male gender: Of study population / proprtion of all male insurants	51.4% / 10.4%
Age (years): Mean study population / mean female / mean male	70.0 / 71.8 / 68.2
Proportion of study population < 40	1.5%
Proportion of study population > =40 < 60	18.0%
Proportion of study population > =60 < 80	58.1%
Proportion of study population > =80	22.4%
Time since diagnosis	
5 years and more	72.3%
4 years	7.5%
3 years	5.9%
2 years	5.7%
1 year	5.1%
Incident diabetes diagnosis 2015	3.6%
Morbidity	
Hypertension	84.9%
Lipometabolic disorder	59.4%
At least one macrovascular event^a^	14.3%
At least one microvascular event^a^	1.7%
T2D related hospitalization^a^	3.4%
Glucose-lowering pharmacotherapy	69.9%
Total non-insulin monotherapy	28.0%
Metformin monotherapy	23.0%
Sulfonylurea monotherapy	2.2%
DPP4 inhibitor monotherapy	2.1%
GLP-1 receptor agonist monotherapy	0.1%
SGLT-2 inhibitor monotherapy	0.1%
Glinide monotherapy	0.4%
Glucosidase inhibitor monotherapy	0.1%
Combination of two non-insulin glucose-lowering drugs	11.7%
Combination of Metformin and Sulfonylurea	3.3%
Combination of Metformin and DPP4	6.6%
Combination of three or more non-insulin glucose-lowering drugs	3.5%
Insulin therapy	26.6%
Insulin only	12.3%
Insulin plus at least one non-insulin glucose-lowering drug	14.3%

^a^Macrovascular events being defined as hospitalization due to stroke (ICD-10 I60.−/I61.−/ I62.−/I63.−/I64.-) or acute myocardial infarction (ICD-10 I21.-) or congestive heart failure (CHF) (ICD-10 I50.-) or coronary revascularizations (OPS 5–361/5–362/5–363) or percutaneous transluminal vascular interventions and stent implantations (OPS 8–836/8–837/8–84) or peripheral vascular disease (ICD-10 I73.9) or angina pectoris (ICD-10 I20.-). Microvascular events being defined as hospitalization due to amputation of the lower extremities (procedural code during hospitalisation: 5–864/5–865) or vitrectomy (procedural code during hospitalisation: 5–158/5–159) or chronic kidney disease, stage 5 (ICD-10 N18.5)

More than 70% of the study population had their T2D diagnosis for at least 5 years. The proportion of newly incident T2D diagnoses in 2015 was 3.6% of all persons with T2D in the sample.

A comorbid diagnosis of hypertension (I10) was found in 84.9% of all persons with T2D. Lipometabolic disorders (E78) were found in 59.4% of the study population.

About 70% of the study population was treated with glucose-lowering pharmacotherapy, with 28% receiving non-insulin monotherapy. One out of four T2D patients (26.6%) was treated by insulin.

### Diabetes-related cardiovascular disease

Using the narrow CVD definition, a CVD prevalence of 46.7% (151,598 out of 324,708; 95% CI [46.52%;46.86%]) among T2D patients was found between 1 January 2010 and 31 December 2015. (see Table 3). Extrapolated to 70,728,398 persons with SHI in Germany in 2015, the number of T2D patients with a history of CVD is estimated to be 2,765,876.

**Table 3:** Primary analysis: Patients with T2D who also had a CVD diagnosis between 2010 and 2015

Patients documented with T2D who had prevalent CVD	N	Proportion of patients with T2D who had prevalent CVD	95% CI	Mean age [years]
Female	66,810	42.3	(42.06; 42.54)	76.52
Male	84,788	50.84	(50.6; 51.08)	72.14
Total	151,598	46.69	(46.52; 46.86)	74.07

The average age of T2D patients with a history of CVD was higher compared to all prevalent T2D patients (74.07 years vs. 70.0 years in all T2D patients). Female T2D patients had a lower proportion of CVD (42.3%; 95% CI [42.06%;42.54%] vs. 50.84%; 95% CI [50.6%;51.08%]) and were found to be in average older than men (76.52 years vs. 72.14 years).

### Impact of observation period and disease definition on CVD prevalence

The prevalence of CVD among T2D patients ranged from 46.7% (narrow definition; 95% CI [46.52; 46.86]) to 57.1% (wide definition; 95% CI [56.9%; 57.24%]) within the main observation period (2010–2015) (Table 4). The proportion of male and female patients, as well as their mean age, were generally similar across different CVD definitions.

**Table 4:** Impact of observation period and disease definition on resulting figures of comorbid CVD in patients with T2D

Observation intervall	% of T2D patients with CVD					
	Narrow definition		Narrow definition including CHF		Wide definition	
	%	95% CI	%	95% CI	%	95% CI
2010–2015	46.7	(46.52; 46.86)	54	(53.83; 54.17)	57.1	(56.9; 57.24)
2011–2015	45.3	(45.1; 45.44)	52.6	(52.44; 52.78)	55.6	(55.44; 55.78)
2012–2015	43.7	(43.53; 43.87)	51	(50.87; 51.21)	54	(53.81; 54.15)
2013–2015	41.9	(41.75; 42.09)	49.2	(49.03; 49.37)	52	(51.86; 52.21)
2014–2015	39.7	(39.56; 39.9)	46.8	(46.63; 46.97)	49.6	(49.41; 49.75)
2015	36.8	(36.63; 36.96)	43.5	(43.29; 43.63)	46.2	(46.03; 46.38)

Using the broader CVD definition without CHF within the main observation period, while additionally excluding OPS codes, the proportion of selected patients is only marginally reduced from 51.2 to 51.1%. Hence, including OPS codes for revascularization and coronary bypass or stent procedures had only very little impact on the identification of prevalent CVD.

**Fig. 2:**
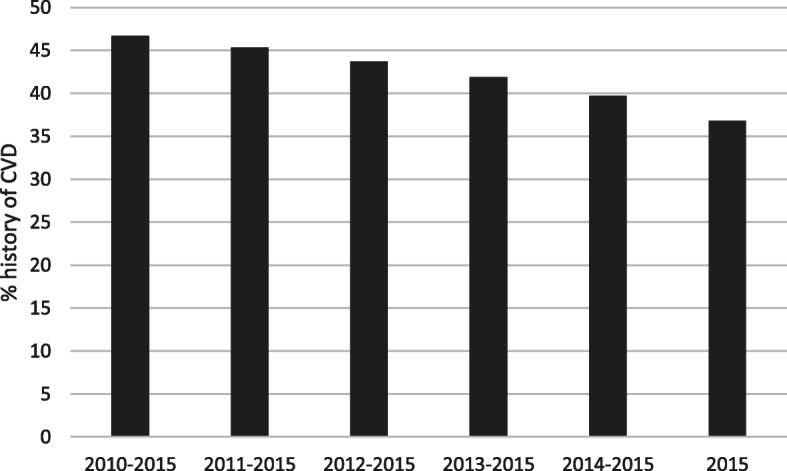
Proportion of CVD in T2D patients. Note: Calculations are based on the “narrow” definition as explained in the Methods section. T2D = Type 2 diabetes, Dx = Diagnosis

The duration of the analyzed observation period had a considerable impact on the proportion of CVD found in T2D patients. The longer the observation interval the higher the proportion of comorbid CVD. Hence, the proportion of T2D patients with diagnoses of CVD was decreasing with the number of observation years. For instance, observing solely the disease history within the T2D index diagnosis year in 2015, the proportion of patients found to have documented CVD decreased to 36.7% [95% CI: 36.63%;36.96%] (Fig. 2, Table 4).

## Discussion

Although T2D is a growing challenge in Germany as is in many other countries, epidemiologic data on specific subpopulations of patients are limited. In particular, few data are available on the prevalence of manifest CVD in patients with T2D. In this study the prevalence of T2D patients with a history of CVD was investigated on a representative health insurance database for Germany. For the time period between 2010 and 2015, 47% of the selected 324,708 individuals with a diagnosis of T2D also had a diagnosis of CVD, resulting in an extrapolated number of 2,765,876 individuals in Germany. CVD was more prevalent in men than in women (51% vs. 42%). The mean age of the study population was 74 years with men being younger than women (72 vs. 77 years).

Sensitivity analyses showed that both the observation period as well as the definition of CVD in terms of included ICD-coded diagnoses had a noticeable impact on the prevalence estimates of CVD. Based on a 6-year observation period – when applying a broad definition of CVD – more than half of the patients with T2D in Germany have a history CVD. However, the prevalence of a history CVD decreases to 36.8% when using a narrow definition of CVD and a one-year interval.

The effect of growing CVD prevalence along with extending observation periods is likely explained by the nature of claims data and could mainly be driven by the following aspect: For reimbursement purposes, it is not always required that all pre-existing diagnoses are repeatedly documented in each consultation. So, even if a patient is continuously in contact with primary care physicians, this does not necessarily mean that all existing medical problems are documented at least once in a year. Thus, analyzing one single year of claims data underestimates prevalence figures of chronic conditions.

Our findings show considerable similarities with previous studies on the same topic. In 2014 and 2015, two claims data-based studies of large German statutory health insurance funds were published [8, 19]. In the first study, Wilke et al. analyzed nearly 400,000 T2D patients in Germany in 2010 and 2011. The mean age of the study population was 72.6 years and thus slightly higher compared to the population of this study (70.0 years). There had been 43.8% women in the study population which is slightly less than what we have found (48.6%). The one-year prevalence of hypertension and lipid disorders were 78.0 and 43.5%, which comes close to the findings of this study being slightly higher with 84.9 and 59.4%, respectively. Overall the study population selected by Wilke et al. showed good accordance with the population selected for this study. Wilke et al. analyzed CVD prevalence for 13 subgroups built up in function of DMP-derived clinical data, namely HbA1c values, blood pressure, and Charlson Comorbidity Index, respectively. Hospitalization with T2D as the main diagnosis was found by Wilke et al. in 3.8% and in the present study in 3.4% of all T2D patients. Wilke found 9.7% of the patients affected by at least one macrovascular event and 1.4% by at least one microvascular event in terms of hospitalization due to defined diagnoses. The according findings in this study were slightly higher with 14.3% of T2D patients being hospitalized due to macrovascular events and 1.7% due to microvascular events. One reason for the higher occurrence of severe events found in this study could be explained by the fact that our observation was done in the year 2015 and thus 5 years after the investigation of Wilke. Our results could reflect growing frequencies of chronic diseases in western civilizations. Another explanation for more macrovascular events in this study could be the fact that Wilke investigated only DMP enrolled patients whereas in this study all T2D patients had been included.

In a second study conducted by Boehme et al. the occurrence of T2D between 2007 and 2010 in the south-west of Germany was analyzed [8]. In 2010 the T2D prevalence was found to be 8.6%. Hypertension was found in 79.9% of patients. The prevalence of coronary heart disease (I20-I22, I24-I25), and stroke (I63-I64) in the study population was 22.9 and 4.1%, respectively, summing up to 27.0% of all T2D patients. This one-year-observation figure is lower than the CVD prevalence figure of 36.8% found in this study, because additional conditions beyond coronary heart disease and stroke had been included in the definition of CVD. As shown in this study the included ICD codes have a significant impact on results. From that perspective, it is coherent that our results including for example additionally arterial occlusive disease revealed more patients with history of CVD in T2D. Furthermore, growing frequencies of chronic diseases in western civilizations could be a driver of higher history of CVD in T2D patients when comparing data from 2010 and 2015.

The considered claims data studies underline that severe cardiovascular damage is frequent in T2D patients. From a clinical perspective, diagnosis and treatment of T2D should be interpreted with a wider scope, including relevant macro- and microvascular diseases. From an epidemiological point of view, it is desirable that population-based data on cardiovascular diseases in T2D patients is collected and evaluated on a larger scale with the aim to get a better picture of the currently ongoing growing burden of chronic diseases in the population. Thus, there is a need for a population-based registration of complications in type 2 diabetes.

### Limitations

We acknowledge some limitations of this study. The structure, strengths, and limitations of German SHI data, in general, have been discussed by others [20]. Limitations resulting from non-uniform or incorrect ICD-10 coding, incomplete diagnoses, lack of standardized criteria of diagnosis as well as structural differences of members of the statutory health insurance funds in Germany apply to this study. These limitations, however, apply to most retrospective studies and certainly to data analysis of every claim. The narrow definition of T2D-related CVD followed the inclusion criteria of an existing clinical trial. Our broad definition of CVD was adding additional ICD codes of other CVDs. Both definitions are arbitrary to a certain degree.

It should be further considered that this study was conducted on a selected sample and not on a complete inventory. As the analyzed sample of 4 million patients was age- and gender-stratified according to official demographic reporting [22], the data can be regarded as representative of the German population. Nevertheless, the extrapolation of results from this study to Germany is afflicted with uncertainty. The prevalence of T2D is strongly associated with higher age, whereas insured persons who change their SHI (and thus are no longer observable without interruption in the database) are predominantly found in the younger population. As Andersohn and Walker show, the SHI data base population is slightly younger (and hence less comorbid) than the overall population, which possibly biases the prevalence of T2D found in our study [18]. However, given that this kind of bias exists at all, we assume it to be quite minor as the validity of recently published studies that build on the same database and address the same research area (T2D), suggest [24].

All results of this study refer only to diagnosed diseases. It should also be considered that there is a considerable number of individuals with undiagnosed T2D. For the identification of CVD in patients with T2D, it was decided to include all individuals with at least one documented ambulatory diagnosis (M1Q) or hospital diagnosis. While demanding at least two documented ambulatory diagnoses (M2Q) represents the standard procedure for claims data analyses, the M1Q criterion was used to reproduce the inclusion criteria in EMPA-REG OUTCOME and maintain transferability of study results to the German population. Inclusion of ambulatory diagnoses without further confirmation may have led to overestimated history of CVD, but as the included CV events are severe and often treated in hospital, the effect of false-positive ambulatory diagnoses is likely to be minor.

### Summary

In this analysis based on a claims database, the prevalence of history of CVD in patients with T2D ranged from 36.3 to 57.1%, depending on the observation period and the definition of CVD. Extrapolated to the population of Germany, there are more than 2,7 million individuals affected. Allowing for sufficiently long observation periods has considerable influence on the detectable prevalence of CVD. Both from a clinical as well as epidemiological point of view it is desirable to collect and report continuously current data on T2D and cardiovascular disease.

## Data Availability

The datasets generated and/or analyzed during the current study are stored at InGef (Institut für Angewandte Gesundheitsforschung Berlin GmbH), www.InGef.de, contact person: Dr. Jochen Walker (Jochen.walker@ingef.de). Permission for data access is granted by ELSEVIER Health Analytics, www.elsevieranalytics.de, contact person: Dr. Sigurd Prieur s.prieur@elsevier.com.

## Abbreviations

ATC: Anatomical Therapeutic Chemical
CHF: Chronic heart failure
CI: Confidence interval
CVD: Cardiovascular disease
EBM: Einheitlicher Bewertungsmaßstab (uniform evaluation standard)
HbA1c: Glycated hemoglobin A1c
HTA: Health technology assessment
ICD: International classification of diseases
ICD-10-GM: ICD-10 German Modification
InGef: Institut für Angewandte Gesundheitsforschung
OPS: Operationen- und Prozedurenschluessel (national classification system of operations and clinical procedures in Germany)
SHI: Statutory health insurance
T2D: Type 2 diabetes mellitus
